# Global leadership initiative on malnutrition criteria using different muscle mass assessment methods in hemodialysis patients: Links to physical performance, quality of life, and clinical outcomes

**DOI:** 10.1038/s41430-026-01803-5

**Published:** 2026-08-06

**Authors:** Ilia Beberashvili, Lawi Suissa, Ada Azar, Racheli Gamliel, Kobi Stav, Shai Efrati

**Affiliations:** 1 Nephrology Division; Yitzhak Shamir Medical Center, Gray Faculty of Medical and Health Sciences, Tel Aviv University, Tel Aviv, Israel, Zerifin, Israel; 2 Internal Department E, Yitzhak Shamir Medical Center, Zerifin, Israel; 3 Nutrition Department, Yitzhak Shamir Medical Center, Zerifin, Israel; 4 Urology Department; Yitzhak Shamir Medical Center, Gray Faculty of Medical and Health Sciences, Tel Aviv University, Tel Aviv, Israel , Zerifin, Israel

**Keywords:** Nutrition, Translational research

## Abstract

**Background/objectives:**

Malnutrition is frequent in maintenance hemodialysis (MHD). We aimed to validate the Global Leadership Initiative on Malnutrition (GLIM) criteria using different muscle mass assessment methods and evaluate their associations with nutritional biomarkers, functional status, quality of life, and mortality.

**Subjects/methods:**

This prospective study included 218 MHD outpatients (63% male; mean age 70.6 ± 12.2 years). Muscle mass was assessed using bioimpedance analysis (BIA), mid-arm muscle circumference (MAMC), and calf circumference (CC). Serum biomarkers, handgrip strength, gait speed, and nutritional scores (Malnutrition Inflammation Score [MIS], Geriatric Nutritional Risk Index [GNRI]) were obtained. Malnutrition was defined as MIS > 10. Associations with SF-36 scales and survival were analysed.

**Results:**

Malnutrition prevalence was 26.1% with BIA-based GLIM, 25.2% with MAMC-based GLIM, and 27.1% with CC. BIA-based GLIM correlated with albumin, MIS, GNRI, phase angle, handgrip strength, and gait speed. MAMC-based GLIM showed similar associations except for gait speed. CC-based GLIM correlated with albumin and GNRI. Agreement with MIS was fair (κ = 0.31–0.35). All GLIM variants showed high specificity but low sensitivity. Only BIA- and MAMC-based GLIM were associated with reduced quality of life. BIA-based GLIM predicted higher mortality (HR 1.70, 95% CI 1.01–2.85), and CC-based GLIM showed a stronger association (HR 2.94, 95% CI 1.41–6.14). MAMC-based GLIM showed a nonsignificant trend.

**Conclusions:**

GLIM demonstrated fair agreement with MIS, high specificity, and meaningful concurrent validity. MAMC- and CC-based GLIM provided practical alternatives to BIA. These findings support further validation of anthropometric GLIM variants in diverse MHD populations.

## Introduction

Malnutrition is common in patients undergoing maintenance hemodialysis (MHD) and is linked to increased morbidity and mortality [1]. Traditional assessments such as the Subjective Global Assessment (SGA) and the Malnutrition Inflammation Score (MIS) integrate subjective, biochemical, and anthropometric data and are widely used in this population [2,3].

To standardize malnutrition diagnosis, the Global Leadership Initiative on Malnutrition (GLIM) proposed criteria that include phenotypic components—unintentional weight loss, low BMI, and low muscle mass—and etiologic components—reduced food intake and inflammation or disease burden [4]. In MHD patients, studies using GLIM criteria have yielded inconsistent findings. Avesani et al. reported low agreement and sensitivity when GLIM was compared with SGA or MIS [5], whereas El Alami et al. found good agreement between GLIM and SGA [6]. Given the high prevalence of cognitive dysfunction in this population [7], an objective tool like GLIM warrants further validation.

Assessing muscle mass, a core phenotypic criterion in GLIM, typically requires advanced tools such as bioelectrical impedance analysis (BIA) or dual-energy X-ray absorptiometry (DEXA), which may not be readily available in dialysis settings. ESPEN supports using anthropometric surrogates—mid-arm muscle circumference (MAMC) or calf circumference—as practical alternatives [8,9]. However, no studies have validated these surrogates for use within GLIM in MHD populations.

This study aimed to validate the GLIM criteria for diagnosing malnutrition in MHD patients using BIA, MAMC, and calf circumference, and to assess their concurrent validity with nutritional markers, functional status, and quality of life, as well as their predictive value for all-cause mortality.

## Subjects & methods

### Study design and participants

This prospective observational study included 218 MHD patients from two prospective cohorts at our institution, both approved by the local ethics committee (ASF-0718 and 0116-23-ASF). The first cohort (*n* = 112) was recruited between January and May 2019 [10]; the second (*n* = 142) between January and May 2023. To avoid duplication, 36 patients who had participated in both cohorts were excluded from the second cohort and included only from the first. Participants were followed from study enrollment until death, kidney transplantation, transfer to peritoneal dialysis, transfer to another dialysis facility, or the administrative censoring date (October 2024), whichever occurred first. Median follow-up duration was 7 months (IQR 6–13.8 months). Survival time was calculated from the date of study enrollment to death or censoring.

Inclusion criteria were age ≥18 years, ≥8 weeks of MHD treatment, and written informed consent. Exclusion criteria included active infection, malignancy, autoimmune disease, chronic use of immunosuppressants, or physical deformities.

Patients underwent thrice-weekly 4-h dialysis using vascular access. Dialysis adequacy was assessed by single-pool Kt/V. Dietary protein intake was estimated via normalized protein catabolic rate (nPCR), derived from urea kinetics [11]. Residual kidney function (RKF) was measured using 48-h urine collections with creatinine and urea clearance [12].

### Malnutrition assessment

Nutritional status was assessed by MIS, and GLIM criteria, while GNRI was calculated as an index of nutritional risk.

GNRI, recommended by the KDOQI guidelines as a screening tool among composite nutritional indices for CKD [13], was calculated as:

GNRI = (14.89 × albumin [g/dL]) + (41.7 × weight/ideal weight), with ideal weight calculated using sex-specific Lorentz equations [14].

MIS includes nutritional history, physical exam, BMI, and labs [3]. MIS has shown strong predictive validity [3] and is effective for monitoring nutritional status in MHD patients [15]. A score >10 defined malnutrition, based on a Spanish multicenter study [16] and ROC analysis in our cohort (Supplementary Fig. 1). Because no universally accepted gold standard for diagnosing malnutrition exists in maintenance hemodialysis patients, we used the Malnutrition–Inflammation Score (MIS) as an external reference criterion. MIS is one of the most extensively validated nutritional assessment tools in dialysis populations and is strongly associated with hospitalization, inflammation, morbidity, and mortality [3,5].

GLIM criteria included ≥1 phenotypic and ≥1 etiologic criterion [4]. We did not perform initial malnutrition screening, as all MHD patients were considered at high risk. The etiologic criterion of reduced intake was assessed using cohort-specific dietary assessment methods. In the first cohort, dietary intake was evaluated using 3-day food diaries including a dialysis day, a non-dialysis day, and a weekend day as previously described [10]. In the second cohort, dietary intake was assessed by 24-h dietary recall obtained by an experienced dietitian during a face-to-face interview and referring to intake on the preceding day. Dietary data were analyzed using computerized nutritional software (Zameret), adapted for the Israeli population, and calculated intake was normalized to ideal body weight according to European Best Practice Guidelines [12]. Reduced intake was defined as energy intake at least 50% below age-specific recommended norms [12]. Inflammation was defined as CRP > 10 mg/L [17].

Muscle mass was assessed using BIA, MAMC, and calf circumference. In accordance with GLIM recommendations, BIA-derived appendicular skeletal muscle mass index (ASMI) was considered the primary phenotypic criterion for the diagnosis of malnutrition, while MAMC- and calf circumference-based cutoffs were evaluated as alternative phenotypic markers. BIA was performed post-dialysis using Nutriguard-M (cohort 1) or BCM (cohort 2), with patients supine for 5 min prior to measurement, following clinical recommendations for bioelectrical impedance analysis [18]. Phase angle (PhA) was calculated as:

$${\rm{PhA}}={\rm{arctangent}}({\rm{Xc}}/{\rm{R}})\times (180/{\rm{\pi }})$$

Although earlier recommendations and manufacturers’ guidance advised caution regarding BIA use in patients with cardiac implantable electronic devices, more recent prospective studies suggest that clinical-grade BIA can be performed safely in this population without clinically significant device interference or arrhythmias [19,20].

Appendicular skeletal muscle mass (ASMM) was estimated using the Sergi equation [21] and normalized by height² (ASMI). Malnutrition cutoffs: ASMI < 7.0 kg/m² (men), <5.7 kg/m² (women) [9].

MAMC was calculated as:

MAMC = mid-arm circumference − 0.314 × triceps skinfold thickness (TSF) with malnutrition defined as <22.5 cm (men) or <20.5 cm (women) [22].

Calf circumference was measured at the point of the largest calf girth using a non-elastic tape, with patients standing upright and weight evenly distributed on both feet. In accordance with GLIM guidelines, low muscle mass based on calf circumference was defined using sex-specific cutoff values: <33 cm (men) or <32 cm (women) [9].

These choices were further supported by ROC curve analyses, which tested the discriminative ability of MAMC and calf circumference for sex-specific ASMI cutoffs in our population (Supplementary Fig. 2).

Anthropometric assessments (body weight, MAMC, and calf circumference) were performed post-dialysis, when patients were at their dry weight.

### Functional status and QoL

Handgrip strength (HGS) was measured using a Harpenden dynamometer, recording the best of three trials in each arm. Gait speed was evaluated as the faster of two timed six-meter walks. This approach was chosen to reduce the influence of initial hesitation or familiarization and to better capture each participant’s best steady walking performance [23]. This distance was chosen to ensure that participants reached a steady walking pace, reducing the influence of acceleration and deceleration phases. An additional 1-meter acceleration zone was used before the timed 6-meter segment. Handgrip strength and gait speed measurements were conducted before the dialysis session to minimize the impact of post-dialysis fatigue and hemodynamic instability. Quality of life was assessed using the SF-36 questionnaire, validated in MHD patients [24]. Comorbidity burden was measured using the dialysis-specific index by Liu et al. [25].

### Laboratory parameters

Mid-week predialysis labs included albumin (bromocresol green), creatinine, urea, CRP (high-sensitivity assay), and lipid panel, using standard automated analyzers.

### Power and statistical analysis

Post hoc power analysis using G*Power 3.1.9.7 confirmed 98.6% power to detect diagnostic performance differences. Normally distributed data were expressed as mean ± SD; skewed variables as median (IQR); categorical data as n (%). T-tests, Mann-Whitney U, and chi-square tests were used as appropriate.

ROC curves defined optimal MAMC and calf circumference cutoffs. Sensitivity and specificity were calculated using 2×2 contingency tables. Positive and negative predictive values (PPV and NPV, respectively) were calculated to assess the diagnostic accuracy of the tests. Additionally, positive and negative likelihood ratios (PLR and NLR, respectively) were computed to determine how much the odds of the disease increase or decrease when a test result is positive or negative, respectively. Agreement was assessed using Cohen’s kappa. Differences between κ coefficients derived from the same participants were evaluated using bootstrap comparison of dependent κ coefficients. McNemar’s test compared paired proportions.

Multivariable logistic regression was used to examine whether GLIM-defined malnutrition was associated with clinically relevant differences across nutritional, functional, body-composition, and quality-of-life domains, as part of the concurrent validation of the GLIM criteria. In these models, GLIM diagnosis (malnutrition: yes/no) was entered as the independent variable, and the clinical parameter of interest was entered as the dependent variable. Logistic regression requires a binary dependent variable; therefore, continuous variables were dichotomized at the median of their distribution. This approach is consistent with prior GLIM validation studies. Cox proportional hazards regression was used to examine the association between GLIM-defined malnutrition and all-cause mortality. Both multivariable logistic regression and Cox regression models were adjusted using the same prespecified clinically relevant covariates: age, sex, dialysis vintage, diabetes, comorbidity index, residual kidney function, and Kt/V.

SPSS v29.0 was used for all analyses.

## Results

The participants had an average age of 70.6 years (± 12.2 years), with 63% being male and 63% diagnosed with diabetes. Over half of the participants received dialysis via an arterial-venous (A-V) fistula or graft, while 45.9% were treated through permanent central catheters. The average Kt/V, a measure of dialysis adequacy, was 1.42 ( ± 0.31) (Table 1). In the study population, 57 patients (26.1%) were identified as malnourished according to the GLIM criteria using BIA-derived ASMI as the primary phenotypic marker (Table 1), with ASMI representing the most frequently met phenotypic criterion in this diagnostic framework (Supplementary Fig. 3). Using the GLIM criteria with MAMC as a surrogate for muscle mass, 25.2% of the patients were identified as malnourished, while the GLIM criteria with calf circumference as a surrogate for muscle mass indicated a slightly lower malnutrition rate of 27.1% (data not shown). Patients diagnosed with malnutrition according to the GLIM criteria were older and exhibited a lower prevalence of diabetes. They lacked residual kidney function and had higher Kt/V values. These patients also showed reduced handgrip strength (in both genders) and slower gait speed, along with lower levels of serum albumin, creatinine, and uric acid. Additionally, they had elevated CRP levels, lower BMI, LBMI, and phase angle, as well as higher MIS and lower GNRI scores, as anticipated (Table 1).

**Table 1:** Demographic, clinical and laboratory parameters of the study population according to GLIM criteria for malnutrition.

Variable	All (*n*=218)	GLIM criteria		*P* value
		Normal nutrition (*n*=161)	Malnutrition (*n*=57)	
**Demographic and clinical parameters**				
Age (y)	70.6±12.2	69.5±12.3	73.8±11.2	0.02
Gender (male) n(%)	138 (63.3)	99 (61.5)	39 (68.4)	0.43
Vintage (months)	29.0 (14.8-48.0)	28.0 (15.0-46.0)	33.0 (12.0-57.0)	0.47
DM n(%)	138 (63.3)	109 (67.7)	29 (50.9)	0.03
Vascular access				0.55
A-V Fistula n(%)	96(44.0)	72(44.7)	24 (42.1)	
A-V Graft n(%)	22(10.1)	18 (11.2)	4 (7.0)	
Permcath n(%)	100 (45.9)	71 (44.1)	29 (50.9)	
Smoker n(%)	41 (18.8)	30 (18.6)	11 (19.3)	0.99
Kt/V	1.41±0.29	1.39±0.28	1.48±0.31	0.04
nPCR (g/kg/d)	1.00±0.25	1.00±0.24	0.97±0.28	0.43
RKF (ml/min/1.73m2)	0.0 (0.0-1.86)	0.2 (0.0-2.0)	0.0 (0.0-0.4)	0.07
Comorbidity index	5 (3-7)	5 (3-7)	6 (4-8)	0.16
Gait speed (sec)	8.5 (5.6-16.3)	7.6 (5.4-14.9)	11.2 (6.2-19.3)	0.04
Handgrip strength (kg)				
Men	20.3 (16.0-27.0)	20.3 (16.0-27.0)	18.0 (11.6-23.6)	0.001
Women	11.3 (6.8-15.8)	11.3 (2.0-9.0)	7.5 (4.5-11.8)	0.03
**Laboratory characteristics**				
Hb (g/dL)	10.8±1.5	10.8±1.5	10.5±1.5	0.20
Albumin (g/dL)	3.73±0.38	3.78±0.35	3.56±0.40	<0.001
Creatinine (mg/dl)	7.03±2.26	7.23±2.34	6.42±1.92	0.02
Uric acid (mg/dl)	5.36±1.24	5.46±1.29	5.04±1.10	0.03
CRP (mg/L)	7.5 (2.8-17.0)	6.0 (2.3-12.1)	16.8 (10.9-31.4)	<0.001
**Body composition**				
BMI (kg.m2)	27.0±5.4	27.9±5.3	24.3±4.8	<0.001
TSF (mm)	19.0 (14.0-28.0)	19.3 (14.0-28.0)	19.5 (12.9-25.0)	0.39
MAMC (cm)	23.3±3.6	24.0±3.6	21.5±2.8	<0.001
Calf circumference (cm)	33.8±4.8	34.3±4.7	32.3±4.7	0.007
ASMI (kg/m2)	6.9±1.4	7.2±1.4	6.0±1.1	<0.001
LBMI (kg/m2)	5.2 (3.9-16.7)	5.4 (4.1-18.6)	4.6 (3.2-14.0)	0.01
FMI (kg/m2)	8.9 (6.1-12.7)	9.3 (6.2-13.3)	8.2 (5.4-11.2)	0.07
ECW/TBW	0.45±0.06	0.45±0.06	0.44±0.06	0.67
Phase Angle (O)	4.5±1.3	4.7±1.3	4.1±1.3	0.008
**Nutritional scores**				
MIS	7.0 (5.0-10.0)	6.0 (4.0-9.0)	10.0 (9.0-13.0)	<0.001
GNRI	106.7 (99.8-114.7)	108.7 (101.9-116.0)	98.3 (91.3-105.9)	<0.001

Continuous variables are expressed as mean ± SD or median with interquartile range (Q1 to Q3) for non-normally distributed data, and categorical variables are expressed as a percentage.

*DM* diabetes mellitus, *nPCR* normalized protein catabolic rate, *CRP* C-reactive protein, *BMI* body mass index, *TSF* triceps skinfold thickness, *MAC* mid-arm circumference, *MAMC* mid-arm muscle circumference calculated, *ECW/TBW* extracellular water to total body water ratio, *FMI* fat mass index, *LBMI* lean body mass index, *ASMI* appendicular skeletal muscle mass index, *MIS* malnutrition-inflammation score, *GNRI* geriatric nutritional risk index.

As part of the ongoing study on the concurrent validation of GLIM criteria using different surrogates for muscle mass, a logistic regression analysis was conducted (Table 2). This revealed significant associations between GLIM criteria based on BIA measurements and gait speed, handgrip strength in both sexes, serum albumin, fat mass index (FMI), phase angle, as well as MIS and GNRI. After multivariable adjustments for age, gender, dialysis vintage, diabetes status, comorbidity index, residual kidney function, and Kt/V, the GLIM criteria remained significantly associated with gait speed, LBMI, MIS, and GNRI. GLIM, when using MAMC as a surrogate for muscle mass, showed similar associations in the univariate regression analysis, except for the lack of associations with gait speed. Additionally, after multivariate adjustments, the association with LBMI was not observed (Table 2). GLIM using calf circumference as a measure of muscle mass demonstrated fewer associations with various nutritional markers compared to GLIM incorporating ASMI calculated from BIA or MAMC measurements. After multivariate adjustments, significant associations were found only with albumin and GNRI (Table 2).

**Table 2:** Associations of GLIM Criteria Using Different Muscle Mass Measurement Methods with Selected Nutritional, Clinical, Laboratory, and Body Composition Markers Based on Univariate and Multivariate Logistic Regression Analyses.

Variable	GLIM (BIA-based)		GLIM (MAMC-based		GLIM (Calf Circ-based)	
	raw	adjusted	raw	adjusted	raw	adjusted
	OR (95% CI)	OR (95% CI)	OR (95% CI)	OR (95% CI)	OR (95% CI)	OR (95% CI)
Gait speed (sec)<8.52	**0.45 (0.24-0.87)**	**0.36 (0.15-0.87)**	0.56 (0.30-1.07)	0.55 (0.23-1.30)	0.57 (0.28-1.17)	0.65 (0.25-1.70)
Handgrip strength (kg)						
Men>20.3	**0.43 (0.19-0.87)**	0.70 (0.27-1.87)	**0.30 (0.13-0.70)**	0.55 (0.21-1.57)	**0.29 (0.11-0.72)**	0.44 (0.13-1.45)
Women>11.3	**0.30 (0.09-0.99)**	0.28 (0.07-1.08)	0.86 (0.27-2.69)	0.71 (0.21-2.41)	0.28 (0.07-1.10)	0.23 (0.05-1.03)
Albumin (g/dL)>3.75	**0.46 (0.25-0.87)**	0.60 (0.29-1.21)	**0.34 (0.18-0.65)**	**0.41 (0.20-0.84)**	**0.34 (0.17-0.70)**	**0.39 (0.17-0.88)**
Creatinine (mg/dl)>6.8	0.60 (0.32-1.12)	0.57 (0.26-1.26)	0.66 (0.36-1.24)	0.61 (0.28-1.35)	0.88 (0.45-1.73)	0.99 (0.43-2.34)
Uric acid (mg/dl)>5.2	0.66 (0.36-1.22)	0.68 (0.34-1.35)	0.73 (0.39-1.35)	0.72 (0.36-1.44)	1.00 (0.51-2.00)	1.18 (0.55-2.52)
nPCR (g/kg/d)>0.98	0.78 (0.42-1.47)	0.75 (0.37-1.51)	1.01 (0.54-1.91)	1.00 (0.49-2.04)	0.84 (0.42-1.68)	0.69 (0.31-1.51)
LBMI (kg/m^2^)>5.2	0.59 (0.30-1.04)	**0.45 (0.22-0.92)**	0.58 (0.31-1.09)	0.52 (0.26-1.05)	1.05 (0.53-2.08)	1.11 (0.51-2.39)
FMI (kg/m^2^)>8.9	**0.49 (0.26-0.93)**	0.71 (0.34-1.49)	**0.46 (0.24-0.88)**	0.63 (0.30-1.38)	0.57 (0.29-1.15)	0.86 (0.37-1.96)
Phase angle (o)>4.4	**0.44 (0.24-0.84)**	0.51 (0.24-1.11)	**0.49 (0.26-0.93)**	0.64 (0.30-1.38)	0.69 (0.35-1.35)	0.83 (0.36-1.92)
MIS<7	**0.14 (0.06-0.32)**	**0.16 (0.06-0.41)**	**0.20 (0.09-0.42)**	**0.23 (0.10-0.54)**	**0.23 (0.10-0.52)**	0.41 (0.16-1.02)
GNRI>106.7	**0.17 (0.08-0.36)**	**0.24 (0.11-0.55)**	**0.20 (0.10-0.41)**	**0.26 (0.12-0.57)**	**0.19 (0.09-0.43)**	**0.37 (0.15-0.88)**

Logistic regression models were constructed with GLIM criteria using different muscle mass assessment methods as independent variables. The variables of interest, listed in the left column of the table, were used as dependent variables, and their values are expressed as being above the median of their respective distributions.

*Adjusted for age, gender, dialysis vintage, DM status, comorbidity index, RKF and Kt/V.

Significant associations at a level of *P* < 0.05 are highlighted in bold.

*DM* diabetes mellitus, *nPCR* normalized protein catabolic rate, *MAMC* mid-arm muscle circumference calculated, *FMI* fat mass index, *LBMI* lean body mass index, *MIS* malnutrition-inflammation score, *GNRI* geriatric nutritional risk index.

**Fig. 1:**
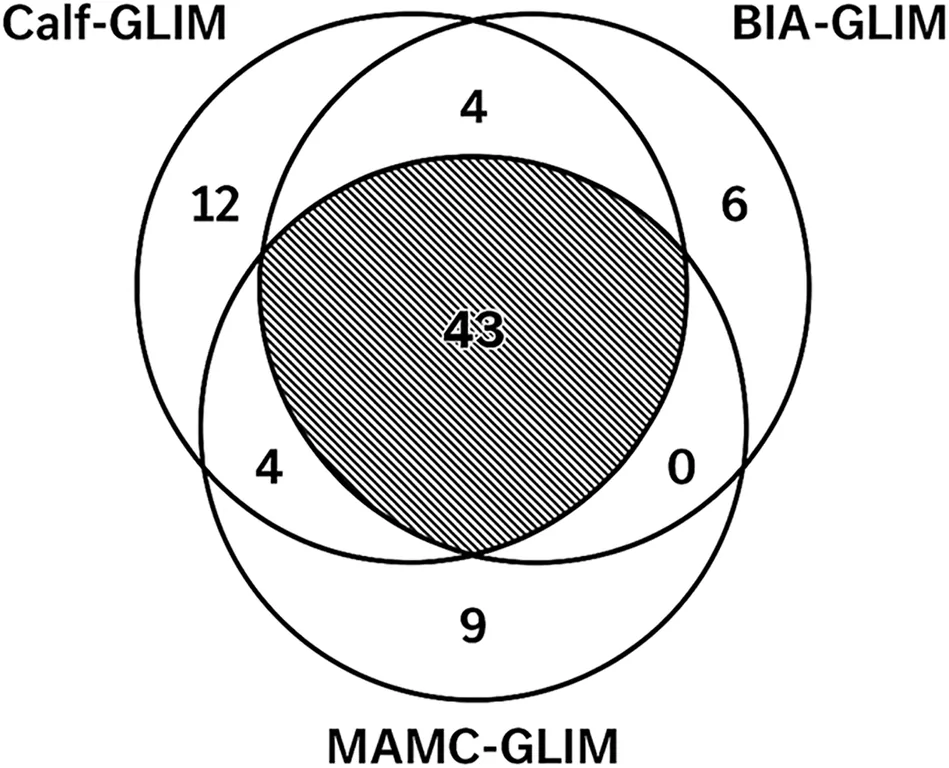
Overlap of malnutrition diagnosis according to GLIM criteria using three muscle-mass assessment methods (BIA, mid-arm muscle circumference, and calf circumference). The Venn diagram illustrates the number of patients identified as malnourished by each GLIM variants. A total of 57 patients were classified as malnourished according to the BIA-based GLIM definition, 56 according to the MAMC-based definition, and 59 according to the calf circumference-based definition. Of these, 43 patients were identified as malnourished by all three GLIM variants. Only a small number of patients were uniquely classified as malnourished by a single method (6 by BIA alone, 9 by MAMC alone, and 12 by calf circumference alone), while few patients were classified by any pair of methods without overlap across all three (4 by BIA + MAMC, 4 by BIA+calf circumference, and 0 by calf circumgerence+MAMC). These patterns demonstrate substantial concordance across the muscle-mass assessment methods used for GLIM phenotyping.

We further explored the ability of GLIM criteria, using different muscle mass surrogates, to replicate the malnutrition diagnosis rate defined by an MIS score above 10, using the kappa statistic (Supplementary Table 1). The κ score indicated fair agreement between malnutrition diagnoses based on MIS and GLIM criteria across all three muscle-mass assessment methods, with numerically lower agreement for the calf circumference-based GLIM than for the BIA- and MAMC-based GLIM (Supplementary Table 1); however, bootstrap comparison of the dependent κ coefficients showed no statistically significant differences between methods. There was substantial agreement between the BIA-based GLIM criteria and the two GLIM variants incorporating anthropometric muscle-mass assessments (Fig. 1). Agreement was excellent for both comparisons, with the κ coefficient slightly higher for MAMC-based GLIM (κ = 0.77, 95% CI: 0.67–0.86) than for calf circumference-based GLIM (κ = 0.74, 95% CI: 0.63–0.83), reflecting the robustness and internal consistency of GLIM phenotyping across measurement approaches.

McNemar’s test revealed no significant difference between malnutrition diagnoses based on an MIS score above 10 and the GLIM criteria using different muscle mass surrogates (Supplementary Table 2). Likewise, when comparing GLIM variants based on anthropometric measurements to those based on BIA-derived ASMI, no statistically significant differences were observed (Supplementary Table 2).

Sensitivity analyses comparing BIA-based, MAMC-based, and calf circumference-based GLIM criteria with MIS > 10, as well as among GLIM methods using anthropometric versus BIA-based standards, are shown in Table 3. All three GLIM variants (BIA-based, MAMC-based, and calf circumference-based) showed low sensitivity but high specificity in predicting malnutrition diagnosed by MIS. This suggests GLIM may under-diagnose malnutrition compared to MIS but is reliable in ruling out malnutrition. The low positive predictive value and high negative predictive value further indicate that GLIM criteria are more accurate in confirming the absence of malnutrition than identifying its presence. Compared to BIA-derived GLIM, the MAMC-based GLIM showed higher sensitivity and specificity for predicting malnutrition than the calf circumference-based GLIM (Table 3).

**Table 3:** Sensitivity Analysis Comparing GLIM Criteria for Malnutrition Using BIA-Based, MAMC-Based, and Calf Circumference-Based Methods Against the Malnutrition-Inflammation Score (MIS >10), and Agreement Between Anthropometric-Based and BIA-Based GLIM Criteria.

Variable	Sensitivity	Specificity	PPV	NPV	PLR	NLR
	95% CI	95% CI	95% CI	95% CI	95% CI	95% CI
**MIS>10 as a reference**						
GLIM (BIA) criteria	56.8	82.5	47.2	87.4	3.25	0.52
	41.0-71.7	75.7-88.0	33.3-61.4	81.0-92.3	2.1-5.0	0/4-0.7
GLIM (MAMC) criteria	54.6	81.9	45.3	86.8	3.01	0.56
	38.5-69.6	75.0-87.5	31.6-59.6	80.3-91.7	2.0-4.6	0.4-0.8
GLIM (Calf Circ) criteria	37.2	84.4	39.0	83.3	2.38	0.74
	23.0-53.3	77.8-89.6	24.2-55.0	76.7=88.7	1.4-4.0	0.6-0.9
**GLIM (BIA) criteria as a reference**						
GLIM (MAMC) criteria	83.6	94.5	83.6	94.5	15.2	0.2
	71.2-92.2	89.8-97.4	71.2-92.2	89.8-97.4	7.9-28.9	0.1-0.3
GLIM (Calf Circ) criteria	57.4	92.4	72.1	86.3	7.51	0.46
	43.2-70.8	87.0-96.0	56.1-84.8	80.1-91.1	4.2-13.6	0.3-0.6

*GLIM* global leadership initiative on malnutrition, *BIA* bioelectric impedance analysis, *MAMC* mid-arm muscle circumference calculated, *Calf Circ* calf circumference, *PPV* positive predictive value, *NPV* negative predictive value, *PLR* positive likelihood ratio, *NLR* negative likelihood ratio.

Malnourished patients identified by BIA-based and MAMC-based GLIM reported significantly lower scores in the mental and physical health dimensions, general health, role physical, and functionality scales of the SF-36 QoL questionnaire (Table 4). The calf circumference-based GLIM was only significantly associated with the physical function scale (Table 4).

**Table 4:** Quality of Life Assessed by SF-36 Questionnaire Dimensions and Scales in the Study Population, Grouped by Presence of Malnutrition According to GLIM Criteria Using Different Muscle Mass Assessment Methods.

Variable	GLIM (BIA-based)		GLIM (MAMC-based)		GLIM (Calf Circ-based)	
	Normal nutrition	Malnutrition	Normal nutrition	Malnutrition	Normal nutrition	Malnutrition
**SF-36 dimensions**						
Mental health	**47 (29-64)**	**36 (25-49)**	**47 (29-64)**	**33 (25-49)**	43 (29-63)	34 (20-55)
Physical health	**50 (32-67)**	**37 (28-54)**	**50 (33-67)**	**36 (28-56)**	47 (32-67)	37 (29-58)
**SF-36 scales**						
Body pain	88 (38-100)	75 (25-100)	94 (38-100)	75 (13-100)	88 (31-100)	63 (13-100)
General health	**36 (25-55)**	**30 (20-40)**	**35 (25-55)**	**35 (20-40)**	35 (25-50)	35 (21-45)
Mental health	70 (55-86)	65 (40-80)	70 (55-85)	65 (40-81)	70 (53-85)	70 (40-85)
Physical function	**50 (16-75)**	**23 (6-54)**	50 (13-75)	30 (10-60)	**50 (13-75)**	**30 (3-53)**
Role emotional	50 (17-75)	25 (17-65)	**50 (17-83)**	**25 (8-58)**	42 (17-75)	42 (17-67)
Role physical	**25 (6-56)**	**19 (0-30)**	**25 (0-56)**	**19 (0-31)**	25 (0-56)	22 (0-36)
Functionality	**50 (13-91)**	**25 (0-75)**	**50 (13-100)**	**25 (0-75)**	50 (13-88)	38 (0-75)
Vitality	38 (25-55)	31 (19-50)	**38 (25-56)**	**28 (19-45)**	38 (25-50)	25 (19-50)

The variables are presented as medians with interquartile ranges due to their skewed distribution. The normal nutrition and malnutrition groups are compared using Mann-Whitney rank-sum tests. Significant associations, with a *P*-value of less than 0.05, are highlighted in bold.

*GLIM* global leadership initiative on malnutrition, *BIA* bioelectrical impedance analysis, *MAMC* mid-arm muscle circumference calculated, *Calf Circ.* calf circumference.

During follow-up, 70 deaths occurred over 256.5 patient-years, corresponding to a mortality rate of 27.3 deaths per 100 patient-years. We compared the predictive ability of the three GLIM variants for all-cause mortality. Patients classified as well-nourished by BIA-based GLIM (Supplementary Fig. 4a) and calf circumference-based GLIM (Supplementary Fig. 4b) had better survival rates. For MAMC-based GLIM (Supplementary Fig. 4c), there was only a trend toward better survival for well-nourished patients. Consistently, in Cox regression analyses (Table 5), BIA-based GLIM was associated with increased mortality risk in fully adjusted models (HR 1.70, 95% CI 1.01-2.85), and calf circumference-based GLIM showed an even stronger association (HR 2.94, 95% CI 1.41-6.14). In contrast, MAMC-based GLIM was non-significant across all models.

**Table 5:** Cox proportional hazards models* for all-cause mortality according to GLIM-based malnutrition (incorporating muscle mass estimated by BIA, MAMC, and calf circumference).

GLIM Method	Model	HR	95% CI	P-value
BIA-derived	Univariate	1.99	1.23–3.22	0.005
	Multivariable model 1	1.70	1.01–2.85	0.04
	Multivariable model 2	1.58	0.89–2.79	0.12
MAMC-derived	Univariate	1.57	0.91–2.69	0.10
	Multivariable model 1	1.28	0.71–2.30	0.41
	Multivariable model 2	1.05	0.54–2.05	0.89
Calf circumference-	Univariate	2.42	1.20–4.87	0.01
derived	Multivariable model 1	2.94	1.41–6.14	0.004
	Multivariable model 2	2.83	1.31–6.12	0.008

Model 1: Adjusted for age, sex, diabetes mellitus, comorbidity index, dialysis vintage, and Kt/V.

Model 2: Adjusted for variables in Model 1 plus C-reactive protein (CRP).

*Handgrip strength and gait speed were not included in these models because they were considered functional outcome/validation variables rather than confounders and may lie on the causal pathway between malnutrition and mortality.

## Discussion

Our study demonstrated that the GLIM criteria identified broadly similar proportions of malnutrition regardless of whether muscle mass was assessed using BIA-derived ASMI, MAMC, or calf circumference. All three GLIM variants showed substantial agreement, with comparable κ coefficients and no significant discordance in McNemar’s tests. These findings indicate that anthropometric muscle-mass surrogates - particularly MAMC and calf circumference - may serve as practical alternatives to BIA for GLIM phenotyping in maintenance hemodialysis patients.

GLIM is a relatively recent framework with growing validation data, including in MHD populations [26,27]. In our cohort, GLIM identified malnutrition in 26.1% of patients, comparable to the 25.8% reported by Aziz et al. [26], though lower than the 42% global prevalence reported elsewhere [10,27]. Differences in prevalence may reflect population characteristics, regional factors [27], and the use of different diagnostic tools such as MIS and SGA [10,27].

In our study, all GLIM variants showed low sensitivity but high specificity when MIS was used as the external reference criterion, indicating that some patients classified as malnourished by MIS were not identified by GLIM, whereas those meeting GLIM criteria likely had clinically meaningful nutritional compromise. This discrepancy may partly reflect the different nature of these tools: GLIM is based on predefined objective criteria [4], whereas MIS incorporates a broader and partly subjective clinical assessment [3]. In the maintenance hemodialysis setting, MIS may therefore detect earlier or more nuanced manifestations of malnutrition, while GLIM may preferentially identify more overt or advanced nutritional compromise. Moreover, because cognitive impairment is common in hemodialysis patients [7], MIS-based assessment may not always be feasible or reliable, which may further support the clinical utility of GLIM as a practical, objective, and standardized diagnostic framework.

We found fair agreement between GLIM and MIS-defined malnutrition (MIS > 10), based on the kappa statistic. Our κ value was higher than those reported by Avesani et al. [5] and Karavetian et al. [28], but lower than El Alami et al. [6]. Such variability likely reflects challenges in defining GLIM’s etiologic criteria in MHD patients and the subjective nature of SGA and MIS, which show moderate intraobserver variability [2,15]. While some studies automatically assign a point for GLIM’s etiologic criteria assuming all MHD patients experience inflammation or reduced intake [5], we believe this may overestimate malnutrition risk. Conversely, El Alami et al. [6] used the modified Glasgow Prognostic Score to define inflammation and food intake, yielding a higher κ, though this score is validated primarily in oncology [29]. We applied GLIM criteria as originally proposed [4,17], recognizing that not all MHD patients have reduced intake or inflammation.

The substantial agreement between BIA-based GLIM and anthropometric variants is noteworthy and lacks direct comparison in prior hemodialysis studies. MAMC performed slightly better than calf circumference in both agreement and discriminatory power. To our knowledge, this is the first study to directly compare BIA-based GLIM with anthropometric GLIM variants incorporating MAMC and calf circumference in maintenance hemodialysis patients. This comparative approach may have practical clinical implications, particularly in dialysis settings where BIA is unavailable or difficult to implement routinely.

Regarding comparative validity, BIA-based GLIM showed strong associations with established nutritional markers in MHD: albumin [3,12], creatinine [12], uric acid [30], phase angle [31], HGS [32], MIS [3,15], and GNRI [15,33]. These associations remained significant in multivariable models, particularly for MIS and GNRI - two well-validated composite indices in the MHD setting.

GLIM’s association with health-related quality of life (HRQoL) is supported by links between its components - body composition, BMI, inflammation, and dietary intake - and SF-36 dimensions in MHD patients [3,34–37]. Although limited to cancer and gastrointestinal disease studies [38–41], prior research suggests GLIM-diagnosed malnutrition may predict lower HRQoL. Our study is the first to show this relationship in MHD, particularly with BIA- and MAMC-based GLIM, reinforcing prior findings of strong associations between MAMC and HRQoL [34].

HGS and gait speed are useful functional indicators in dialysis populations [42]. BIA-based GLIM was associated with both, while MAMC and calf circumference showed associations only with HGS in men. This may reflect the limited ability of anthropometric surrogates to capture functional deficits. Similar discrepancies have been seen in sarcopenia studies in elderly Turkish [43] and peritoneal dialysis populations [44], where MAMC predicted HGS in men but not women. These findings may explain our sex-specific results.

Regarding mortality, using individual follow-up times, the incidence rate was 27.3 deaths per 100 patient-years (273 per 1000 patient-years). Although higher than the 15–20 per 100 patient-years typically reported in hemodialysis registries [45], this is consistent with the high-risk clinical profile of our cohort, characterized by advanced age, a high prevalence of diabetes, substantial comorbidity burden, elevated inflammatory markers, and frequent catheter use - factors well-established to increase mortality in hemodialysis patients [46,47]. Therefore, some degree of selection bias cannot be excluded, and the observed mortality rate and prognostic associations of GLIM should be interpreted in the context of a high-risk maintenance hemodialysis population. Accordingly, the generalizability of these findings to younger, healthier, or lower-risk hemodialysis populations may be limited.

In terms of predictive validity, GLIM-defined malnutrition was associated with mortality in partially adjusted models, consistent with the direction reported by Avesani et al. [5]. Calf circumference-based GLIM showed the strongest association across all models, in line with prior evidence identifying calf circumference as an independent predictor of mortality in hemodialysis patients [48]. In contrast, MAMC-based GLIM demonstrated only a non-significant trend in univariate analysis; however, given that MAMC itself has been independently associated with survival in the maintenance hemodialysis population [34], the limited power of the present study may have contributed to the lack of significance. Importantly, these modest mortality associations do not detract from the clinical relevance of our findings, as GLIM was developed primarily as a diagnostic framework for malnutrition rather than a mortality prediction tool. Notably, prior dialysis studies [5,27,49] have similarly reported attenuated or non-significant associations after multivariable adjustment, indicating that variability in GLIM’s prognostic performance is expected and reflects both population heterogeneity and limited event numbers.

Our study has limitations. As an observational, single-center study, causal inference is limited, and generalizability may be affected. The inclusion of two temporally separated cohorts could introduce bias. Dietary assessment methods also differed between cohorts; however, both were prospectively collected, standardized nutritional assessment approaches commonly used in routine clinical research settings [50]. Consistent inclusion criteria, laboratory procedures, assessment tools, and personnel helped minimize this risk. The study was underpowered for survival analysis, which should be addressed in future research.

In summary, GLIM showed fair agreement with MIS-defined malnutrition in MHD patients, with high specificity and negative predictive value but low sensitivity. Despite this, GLIM criteria demonstrated solid concurrent validity through associations with nutritional markers, scores, function, and HRQoL. When BIA is unavailable, anthropometric muscle-mass surrogates - particularly MAMC and calf circumference - provide practical alternatives with comparable validity. Calf circumference may be particularly useful in settings where MAMC measurement is not feasible, such as when both arms have vascular access. Our findings support further validation of GLIM criteria with anthropometric surrogates in diverse dialysis populations.

## Supplementary information

Supplemental Material

## Data Availability

The data analyzed during this study are available from the corresponding author upon reasonable request.
