# Supplementary material for: Global leadership initiative on malnutrition criteria using different muscle mass assessment methods in hemodialysis patients: Links to physical performance, quality of life, and clinical outcomes
Source: Eur J Clin Nutr. 2026 Aug 6;80(10):1079–87. doi: 10.1038/s41430-026-01803-5 (PMC13690562; doi:10.1038/s41430-026-01803-5)
Supplement: Supplementary file 1 [file 41430_2026_1803_MOESM1_ESM.docx]

Supplemental data

***Global Leadership Initiative on Malnutrition Criteria Using Different Muscle Mass Assessment Methods in Hemodialysis Patients: Links to Physical Performance, Quality of Life, and Clinical Outcomes***

Ilia Beberashvili, MD^1^; Lawi Suissa MD^2^, Ada Azar BCs MHA^3^, Racheli Gamliel, BCs^3^, Kobi Stav, MD^4^; Shai Efrati, MD^1^.

^1^ Nephrology Division, Yitzhak Shamir Medical Center, Gray Faculty of Medical and Health Sciences, Tel Aviv University, Tel Aviv, Israel ; Zerifin, Israel

^2^ Internal Department E, Yitzhak Shamir Medical Center, Zerifin, Israel

^3^ Nutrition Department, Yitzhak Shamir Medical Center, Zerifin, Israel

^4^ Urology Department, Yitzhak Shamir Medical Center, Gray Faculty of Medical and Health Sciences, Tel Aviv University, Tel Aviv, Israel ; Zerifin, Israel

**Legends to Figures:**

**Supplemental Figure 1.** ROC Curve Analysis of the Predictive Ability and Determination of Cut-Off Point for Malnutrition-Inflammation Score in Predicting All-Cause Mortality in the Study Population.

**Supplemental Figure 2.** ROC curve analysis of the predictive ability and determination of cut-off points for BIA-derived appendicular skeletal muscle index (ASMI) using sex-specific norms: Midarm muscle circumference (MAMC) for men (a) and women (b), and calf circumference for men (c) and women (d).

**Supplemental Figure 3.** Prevalence of components of etiologic and phenotypic criteria of GLIM in the study population (n=218). Components of etiologic and phenotypic criteria were defined as follows: Reduced food intake as <50% of energy requirements > 1 week; inflammation as CRP>10mg/L; weight loss as >5% within past 6 months; body mass index (BMI) (kg/m^2^) as <20 if < 70 years, or <22 if >70 years and BIA-derived appendicular skeletal muscle mass index (ASMI) (kg/m^2^) as <7.26 for male and <5.25 for female.

**Supplemental Figure 4.** Kaplan-Meier survival curves comparing survival rates between malnourished and normally nourished hemodialysis patients, diagnosed by GLIM criteria using different methods of muscle mass assessment: (a) BIA-based, (b) calf circumference-based, and (c) MAMC-based.

**Supplemental Table 1.** Inter-Reviewer Agreement Between GLIM Criteria Using Different Muscle Mass Assessment Methods and Malnutrition-Inflammation Score Above 10 for Malnutrition Diagnosis According to Weighted Kappa Statistic.

Variable	Weighted Kappa*	95% CI

GLIM (BIA) criteria	0.35	0.22 to 0.50

GLIM (MAMC) criteria	0.33	0.19 to 0.48

GLIM (Calf Circ) criteria	0.31	0.16 to 0.38

Abbreviations: GLIM, global leadership initiative on malnutrition; BIA, bioelectric impedance analysis; MAMC, mid-arm muscle circumference calculated; Calf Circ, calf circumference.

*No statistically significant differences were observed between κ coefficients (BIA vs calf circumference, P=0.42; MAMC vs calf circumference, P=0.69; BIA vs MAMC, P=0.73).

**Supplemental Table 2.** Comparison of GLIM Criteria for Malnutrition Using BIA-Based, MAMC-Based, and Calf Circumference-Based Methods Versus Malnutrition-Inflammation Score (MIS) Above 10, and Between GLIM Methods According to McNemar's Test

Variable	Difference (%)	95% CI	P value

**MIS>10 as a reference**

GLIM (BIA) criteria	4.41	-2.64 to 10.89	0.15

GLIM (MAMC) criteria	4.41	-2.78 to 11.05	0.20

GLIM (Calf Circ) criteria	5.99	-6.34 to 8.19	0.10

**GLIM (BIA) criteria as a reference**

GLIM (MAMC) criteria	0.46	-3.96 to 3.96	1.00

GLIM (Calf Circ) criteria	-0.92	-4.84 to 2.99	0.83

Abbreviations: GLIM, global leadership initiative on malnutrition; BIA, bioelectric impedance analysis; MAMC, mid-arm muscle circumference calculated; Calf Circ, calf circumference.
